# 39 Are you well? An investigation into the health-related behaviours and wellbeing of Dundalk Institute of Technology (DKIT) students. A pilot study

**DOI:** 10.1093/eurpub/ckae114.040

**Published:** 2024-09-26

**Authors:** Sean Kilroy, Sinead O’Connor, Fiona Hackett, Noeleen Gregory, BSc (Hons) Health & Physical Activity Students

**Affiliations:** Dundalk Institute Of Technology, Dundalk, Ireland; Dundalk Institute Of Technology, Dundalk, Ireland; Dundalk Institute Of Technology, Dundalk, Ireland; Dundalk Institute Of Technology, Dundalk, Ireland; Dundalk Institute Of Technology, Dundalk, Ireland

## Abstract

**Introduction:**

For young adults, adjustment into student life can often be a difficult and challenging time with students facing academic demands, social network changes, financial constraints, and the transition to independence. As a result, many college students tend to engage in harmful health related behaviours which may consequently impact their overall wellbeing. Physical inactivity, alcohol abuse, smoking tobacco, and poor dietary habits are considered main contributing factors associated with chronic disease. In addition, risky health related behaviours and poor wellbeing are associated with attendance issues, cognitive decline, and poor academic performance in the student population.

**Aims:**

To examine the health-related behaviours and wellbeing of students attending DKIT.

**Methods:**

A cross-sectional sample of DKIT students over the age of 18 were recruited by convenience sampling through flyers, peer referrals and word-of-mouth. Participants provided informed consent prior to completing a fitness assessment and self-report survey to examine their body mass index (BMI), physical activity levels (IPAQ-SF), sporting participation, sedentary behavior, sleep quality (PSQI), dietary habits, smoking status, and alcohol consumption.

**Results:**

In total, 169 students (52% Males and 45% Females) took part in the study. Of the sample, 32% were overweight or obese, 23% reported being insufficiently active, 27% did not take part in sport while the average sitting time of students on a typical weekday was 6 hours. In addition, 27% consumed alcohol at least once a week, 8% regularly smoke tobacco and 21% currently use E-cigarettes. Regarding dietary habits, only 50% of students had fruit and 45% had vegetables once or more a day. Finally, 56% of students experienced poor sleep quality and over 15% of the participants reported poor wellbeing.

**Conclusion:**

Understanding these risky health related behaviours in the student population may help guide colleges and universities to adopt better health promotion strategies and interventions to increase awareness, further educate and provide additional support and opportunities for the wellbeing of students.

